# Timed Up and Go and Six-Minute Walking Tests with Wearable Inertial Sensor: One Step Further for the Prediction of the Risk of Fall in Elderly Nursing Home People

**DOI:** 10.3390/s20113207

**Published:** 2020-06-05

**Authors:** Fabien Buisseret, Louis Catinus, Rémi Grenard, Laurent Jojczyk, Dylan Fievez, Vincent Barvaux, Frédéric Dierick

**Affiliations:** 1 Centre de Recherche et de Formation (CeREF), Chaussée de Binche 159, 7000 Mons, Belgium; buisseretf@helha.be (F.B.); catinus.louis@outlook.com (L.C.); remi.grenard@gmail.com (R.G.); jojczykl@helha.be (L.J.); dylan.fievez@cerisic.be (D.F.); barvauxv@helha.be (V.B.); 2 Haute Ecole Louvain en Hainaut (HELHa), Chaussée de Binche 159, 7000 Mons, Belgium; 3 Service de Physique Nucléaire et Subnucléaire, UMONS, Research Institute for Complex Systems, 20 Place du Parc, 7000 Mons, Belgium; 4 Centre National de Rééducation Fonctionnelle et de Réadaptation—Rehazenter, Laboratoire d’Analyse du Mouvement et de la Posture (LAMP), 2674 Luxembourg, Luxembourg; 5 Faculté des Sciences de la Motricité, Université catholique de Louvain, 1348 Louvain-la-Neuve, Belgium

**Keywords:** risk of fall, elderly, wearable sensor, gait variability, clinical tests

## Abstract

Assessing the risk of fall in elderly people is a difficult challenge for clinicians. Since falls represent one of the first causes of death in such people, numerous clinical tests have been created and validated over the past 30 years to ascertain the risk of falls. More recently, the developments of low-cost motion capture sensors have facilitated observations of gait differences between fallers and nonfallers. The aim of this study is twofold. First, to design a method combining clinical tests and motion capture sensors in order to optimize the prediction of the risk of fall. Second to assess the ability of artificial intelligence to predict risk of fall from sensor raw data only. Seventy-three nursing home residents over the age of 65 underwent the Timed Up and Go (TUG) and six-minute walking tests equipped with a home-designed wearable Inertial Measurement Unit during two sets of measurements at a six-month interval. Observed falls during that interval enabled us to divide residents into two categories: fallers and nonfallers. We show that the TUG test results coupled to gait variability indicators, measured during a six-minute walking test, improve (from 68% to 76%) the accuracy of risk of fall’s prediction at six months. In addition, we show that an artificial intelligence algorithm trained on the sensor raw data of 57 participants reveals an accuracy of 75% on the remaining 16 participants.

## 1. Introduction

Falls are an inevitable part of aging and their prediction and prevention are of paramount importance to health care. According to the World Health Organization (WHO), falls are the second cause of accidental death and approximately 646,000 people die every year following falls, particularly people over the age of 65. In the European Union (EU), an average of 35,848 elderly people/y (65 and above) are reported to have died from falls [1]. As the population of elderly people in the EU is expected to grow by 60% by 2050, the number of fall-related deaths is expected to increase to almost 60,000/y by 2050, unless additional measures are taken to predict and prevent falls. Additionally, falls result in significant physical and psychosocial costs that have to be incurred by patients and social security. The social security cost for treating fall-related injuries in the EU is estimated to be €25 billion each year [2].

In nursing homes, falls frequency is higher for elderly people than at home with a yearly rate of falls of 50% [3]. This phenomenon is foreseen to increase in the future in view of the population aging. Risk factors for falls in elderly people are higher among nursing home residents compared to home-based individuals [4]. Those factors are usually grouped in three main categories: intrinsic (age, sex, cognitive disorders, eyesight impairment, sarcopenia, multiple medication use, sensory impairment, gait disorders, postural instability and neurodegenerative diseases), behavioral (inappropriate footwear, alcohol use), and environmental (slippery surfaces, poor lighting, worn carpeting) [3,4,5].

It is well-known that the assessment of the risk of falls is better achieved through screening tools designed for that purpose. One of the most well-known tests is the Timed Up and Go (TUG) test [6]. It is considered as the gold standard in fall risk assessment and has numerous advantages. It is simple and easy to describe and to perform, and, therefore, widely used [7]. It is also recommended by the American Geriatric Society and the British Geriatric Society [8]. The typical time to perform the test is around 20–30 s. Unfortunately, this is too short to enable a full assessment of the gait kinematics and more specifically the long-term variability of the stride interval. This is correlated to the risk of fall in home-based older people [9] but requires a time series of a typical duration of 10 min. More recently, the TUG test has been performed with patients equipped with an accelerometer. This allowed an analysis focusing on sit-to-stand and stand-to-sit subtasks, which is useful to identify fall risk in home-based fallers versus healthy controls [10]. The findings of this study show that the TUG test allows successfully identifying 63% of fallers and this is increased by up to 87% in accelerometer-equipped patients.

Another widely used tool in clinical practice that allows the study of gait for a longer period is the six-minute walking test [11]. Similar to the TUG test, the accelerometer-based six-minute walking test has been used in patients with chronic heart failure [12] and chronic obstructive pulmonary disease [13]. To the best of our knowledge, the accelerometer-based six-minute walking test has not been performed in elderly people to assess risk of fall.

Today, the availability of low-cost inertial sensors enables 3D measurement of acceleration and angular speed during human movements. In a recent study, we successfully used a homemade ultralow-cost wearable inertial sensor (DYSKIMOT) to capture the rotational movements of the head during a standardized test [14]. It is part of the DYSKIMOT project to show the relevance of the proposed sensor in a wide variety of clinically relevant situations, and the present study aims at extending the range of applications of the DYSKIMOT sensor. Of course, many other wearable sensors are relevant in gait analysis, e.g., smart socks [15] or instrumented shirts [16]. Our results can also be transposed in principle to other systems using inertial sensors. Here, we used this sensor to capture locomotor movements during the six-minute walking test to increase the predictive power of the TUG test on the risk of falls among elderly nursing home fallers and nonfallers during two sets of measurements at six months interval (TUG+ test). We also present an analysis of kinematic data based on variability assessment and on artificial intelligence (AI) techniques. Current hardware capacities the processing and management of huge datasets required for such tools to converge to a solution. That is why these techniques have gained popularity in recent years and are recognized as efficient tools in fall detection in particular [17].

The main objective of this study is to show that it is possible to design a test identifying nursing home residents presenting a risk of fall, the test’s accuracy being assessed by comparison to their actual falls during six months after the test. A secondary objective is to present three different ways of assessing the risk of falls: a standard clinical test (TUG), a standard clinical test augmented by sensor measurements (TUG+), and an AI algorithm based on sensor measurements only. The first two tests require the presence of a therapist, while the third could be implemented in an autonomous wearable system.

## 2. Materials and Methods

### 2.1. Population

Participants in this survey (Table 1) were at least 65 years old or over and lived in 4 nursing homes in the Charleroi area in Belgium. The experiment protocol was designed according to the Helsinki declaration and was approved by the Bioethical Academic Committee (No. B200-2017-144). Exclusion criteria were as follows: lower limb musculoskeletal or cardio-respiratory disorders preventing a six-minute walking test or major cognitive disorders preventing a full patient’s cooperation. During the experiment, participants who experienced a major illness such as stroke or limb fracture were excluded.

Participants who chose to discontinue their participation, were admitted to hospital, had their medication changed thus preventing the continuation of the experiment or died before the completion of the experiment were also excluded.

Ninety-two individuals started the initial test. Twelve of them were excluded from the survey in view of the above criteria. Seventy-three participants completed the experiment.

### 2.2. Protocol

**Figure 1:**
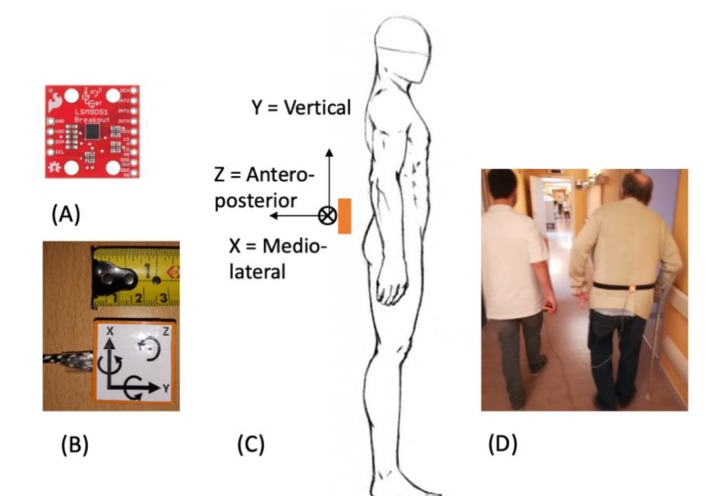
(**A**) The LSM9DS1 sensor (SparkFun) used to record acceleration and angular speed. (**B**) LSM9DS1 sensor within the DYSKIMOT system. Sensor’s cartesian frame is indicated. The curved arrows give rotation’s positive direction. (**C**) Schematic placement of a DYSKIMOT sensor on a patient. The sensor’s cartesian frame matches the walking directions. (**D**) A patient equipped with DYSKIMOT sensor (right) with one experimenter (R.G.) walking aside.

The survey took place between the month of May (t_1_) and the month of November (t_2_) 2018. At t_1_, the TUG test was performed in all participants. Participants sat down with their back against a 46 cm high chair rest. When signaled, participants were asked to get up, walk three meters, turn around and sit down again. Participants then performed the six-minute walking test with the right to take short pauses as required. Measurements were taken by the two same experimenters (L.C. and R.G.), who always walked aside the patient to prevent any fall in case they lost balance. Six-minute tests were performed by walking a typical point-to-point track of 25 m with serial turnarounds. The turnaround points were clearly marked with strong adhesive tape stuck on the floor. During the six-minute walking test, participants were equipped with a homemade sensor collecting several kinematic data. The ultralow-cost sensor, called DYSKIMOT, was presented in a previous survey [14]. It is based on the Magnetic Angular Rate and Gravity (MARG) sensor LSM9DS1 (SparkFun), composed of a 3-axis accelerometer, gyrometer and magnetometer, plus a temperature sensor (Figure 1A,B). It is light (10.44 g) and small enough (3 × 3 cm) to be worn by a patient without any disturbance (Figure 1B). Among other quantities, the MARG sensor measures acceleration, $\vec{a}\left(t\right)$ (in [g], ±16 [g]), and angular velocity, $\vec{\omega }\left(t\right)$ (in °/s, ±2000 °/s) at a sampling frequency of 100 Hz. The data are transmitted to a PC via an Arduino Uno Rev 3 and a USB cable (RS232 serial link) and then transferred to a homemade acquisition software for further analysis. More details can be found in [14], including a comparison between DYSKIMOT and a gold standard optoelectronic sensor. The sensitivity depends on the sensor and on the selected range; detailed information is given in the datasheet (https://www.st.com/en/mems-and-sensors/lsm9ds1.html). For example, the gyrometer sensitivity is 8.75 × 10^−3^ °/s/LSB at the range ±245 °/s, i.e., the range we use in the present study, and the accelerometer sensitivity is 0.322× mg/LSB at the range ±4× g. During the experiment, the DYSKIMOT was positioned in the lumbar region of the individual at the level of the L4 vertebra (Figure 1C,D) in such a way that the sensor’s cartesian frame matched with walking directions.

Between t_1_ and t_2_, each fall of a participant was recorded by the nursing home staff. The fall records were used at t_2_ to categorize participants into fallers and nonfallers. At the end of the survey, 23 fallers and 50 nonfallers were noted. Among the 23 fallers, 17 participants made 1 fall, while 2 participants fell two, three and four times. The lack of “frequent fallers” (more than 1 fall) in our population has led us to consider a binary classification rather than a more detailed description in terms of, say, the number of falls between t_1_ and t_2_.

### 2.3. Data Analysis

**Figure 2:**
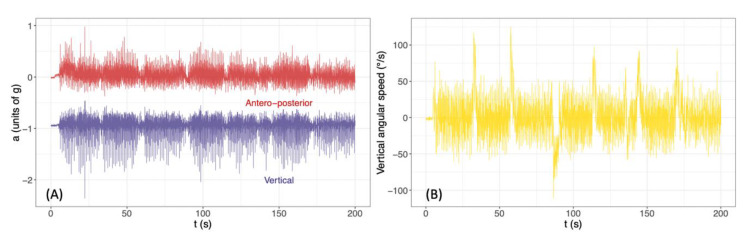
(**A**) Typical traces of anteroposterior ($a_{ap})$ and vertical ($a_{v})$ accelerations measured by the DYSKIMOT during the six-minute walking test. (**B**) Typical trace of vertical angular speeds ($\omega_{v}$) measured by the DYSKIMOT during the six-minute walking test. The regularly spaced peaks correspond to turnarounds made by the participants.

Time series included $\vec{\omega }=\left(\omega_{ml},\omega_{v},\omega_{ap}\right)$ and $\vec{a}=\left(a_{ml},a_{v},a_{ap}\right)$ (2 times 3 components). Typical traces of selected time series are shown in Figure 2. The indices *ml*, *v* and *ap* stand for mediolateral, vertical and anteroposterior, respectively. We recorded for analysis the data at t_1_ and t_2_ of the 73 participants still included at t_2_.

We chose to focus on the assessment of time series variability. As shown in [5], gait variability can be correlated with the risk of fall. Further studies such as [9] have analyzed it using mathematical tools such as fractal dimension and have demonstrated that those tools can discriminate between healthy and disabled individuals, healthy individuals showing a higher fractal dimension. The parameters identified in the present study are the standard deviation (SD) and fractal dimension (D) obtained from the six time series ($\vec{a}$ and $\vec{\omega }$) recorded during the six-minute walking test. Fractal dimension was computed by resorting to the box counting method: If N(ε) is the number of square boxes of side ε necessary to cover the plot of the time series under study, then N(ε) scales as $\varepsilon^{-D}$ when ε→0. D is, therefore, the slope of N(ε) vs. ε in a log–log plot for small values of ε. Computational details about the method we use are presented in [19] and additional mathematical references can be found in [20]. SD and D give complementary information about gait variability: SD provides an indication about the magnitude of the fluctuations while D represents the time series complexity, i.e., smooth (D close to 1) or abrupt (D close to 2) relative changes in successive measurements.

Due to failed normality tests on data, a Mann–Whitney test was performed with a significance level of 0.05 in order to ascertain potential differences between fallers and nonfallers at t_1_.

Three classification algorithms were then defined to classify participants as presenting a risk of fall or not: the TUG test, the TUG+ test and the AI algorithm (see below for more details). Standard tools belonging to binary classification were then used to compare our “diagnostic” (risk of fall or not) to the actual faller or nonfaller status of our participants. Sensitivity ($Se=\frac{TP}{TP+FN}$), specificity ($Sp=\frac{TN}{TN+FP}$), positive ($PPV=\frac{TP}{TP+FP}$) and negative ($NPV=\frac{TN}{TN+FN}$) predictive values, and accuracy ($Acc=\frac{TP+TN}{TP+FP+TN+FN}$) were calculated, with *TP* the true positives, *FP* the false positives, *FN* the false negatives, and *TN* the true negatives. The positive ($LR_{+}=\frac{Se}{1-Sp}$) and negative ($LR_{-}=\frac{1-Se}{Sp}$) likelihood ratios were also computed.

**Figure 3:**
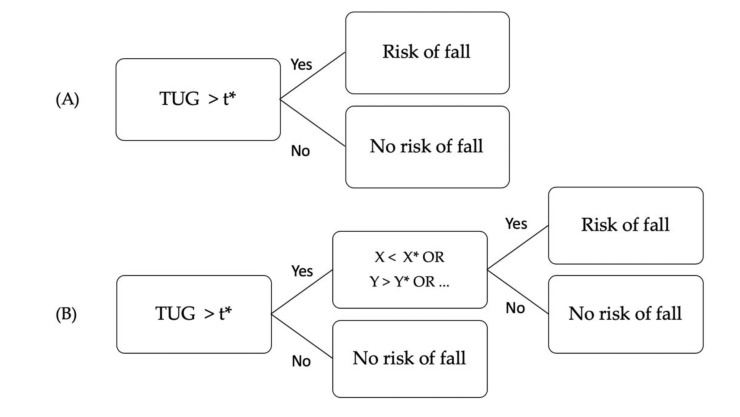
(**A**) Decision tree illustrating the TUG test. T* is the threshold time maximizing Youden’s index. (**B**) Decision tree illustrating the TUG+ test. X, Y, etc., are variability indices and X*, Y*, etc., are threshold values. The selected indices and thresholds are given in Figure 5B.

The TUG test at t_1_ was first analyzed by computing a Receiver Operating Characteristic (ROC) curve. The time maximizing Youden’s index Sp+Se−1 was computed and chosen as the threshold, t*, to separate participants with and without risk of fall. An augmented TUG test (TUG+) was then designed following the decision tree shown in Figure 3. It includes information from the variability indices that showed a significant difference between fallers and nonfallers at t_1_. If an individual is recognized by the clinical test as a faller, then he/she is assessed a second time by one or more kinematic parameters displaying a significant difference between fallers and nonfallers (according to fall recording). Threshold values were chosen after several attempts at designing an augmented TUG test as it provided the best accuracy.

**Figure 4:**
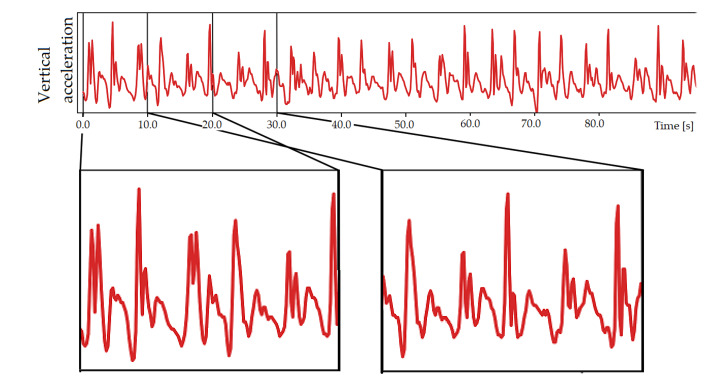
Example of sequence splitting for the train dataset. A duration of 20 s is chosen with 10 s overlapping.

The AI algorithm was designed as follows. The times series related to a participant were divided into fixed-duration windows so that the inputs of our model are fixed-size vectors. Models were trained and tested for different window sizes; the value 20 s was chosen, see Section 3.3 for a justification. Splitting one 6 min time series into smaller ones may also have the advantage of avoiding the AI to learn long-term autocorrelations which are typical of human walk [9,21]. We used a 10 s overlapping during the frame generation of the training dataset (Figure 4) to increase its size and to remove any bias regarding the starting position of the frames. The obtained dataset was divided by test/validation datasets and training as a compromise between having enough fallers data for test/validation and for training. The test/validation is made up of 16 participants (8 fallers and 8 nonfallers randomly chosen) and the training dataset is made up of 57 participants (16 fallers and 41 nonfallers). Models based on Convolutional Neural Network (CNN) [22] have then been trained and tested to find the optimal accuracy on the risk of fall prediction in the test/validation dataset. We have chosen an AI classification based on deep learning (CNN) among other machine learning solutions because of its capacity to extract features by itself. Since TUG and TUG+ methods are based on features we selected (TUG time and variability indices), such a deep learning approach was preferred because it is complementary.

Sigmaplot (v. 11.0) and R (v. 3.5.0) software were used to perform the statistical calculations. AI algorithm was designed by using the standard software Keras over Tensorflow 2.0. R package pROC was used.

## 3. Results

### 3.1. Variability Indices

The variability indices (SD and D) computed from the six time series recorded during the six-minute test are shown in Table 2. The medians are compared and the *p*-values are indicated. Two parameters are significantly different for the fallers and nonfallers: SDa_ap_ is significantly larger for fallers and Da_v_ is significantly smaller. Those parameters will then be selected in the TUG+ test. Note that the fractal dimensions are globally smaller for fallers than for nonfallers.

### 3.2. TUG and TUG+ Tests

**Figure 5:**
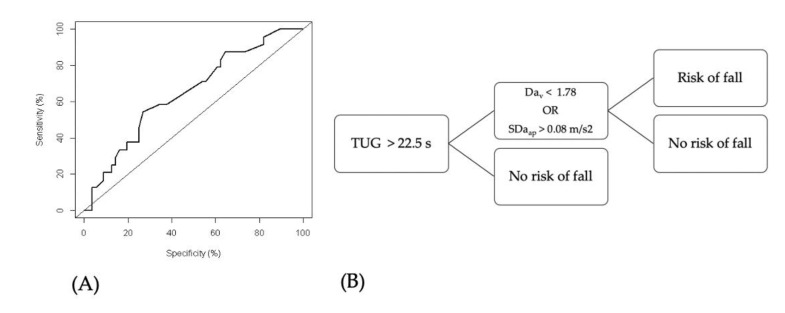
(**A**) ROC curve for TUG test. An area under the curve of 0.621 is obtained, a Youden’s J of 0.256 and a threshold of 22.5 s. (**B**) TUG+ test classification algorithm.

**Figure 6:**
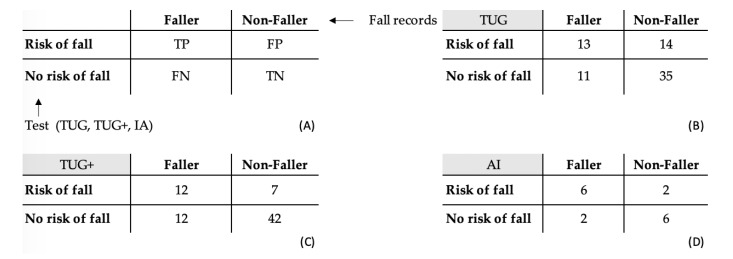
Confusion matrices in our study. (**A**) General definitions. TP = True Positives, FP = False Positives, FN = False Negatives, TN = True Negatives. (**B**) Results for the TUG test. (**C**) Results for the TUG+ test. (**D**) Results for the AI classification. The validation data set has 16 participants.

Fallers perform the TUG test significantly slower than nonfallers. ROC curve and confusion matrix for the TUG test are shown in Figure 5 and Figure 6. Related parameters are shown in Table 3. Youden’s statistics for the TUG test leads to an optimal threshold of 22.5 s. An individual performing the TUG test over that threshold is likely to be considered as presenting a risk of fall in our population.

The threshold of 22.5 s is kept in the TUG+. The SDa_ap_ and Da_v_ thresholds were adjusted in order to maximize the TUG+ test’s accuracy: They are shown in Figure 5. The TUG+ test has better accuracy than the TUG test—an Mc Nemar test performed on the TUG and TUG+ classifications confirm that both tests are significantly different (*p* = 0.013). A detailed comparison between both tests is given in Table 3. The confusion matrix for the TUG+ test is also shown in Figure 6.

We have purposely designed TUG and TUG+ tests in a simple way, like how clinical tests are usually designed. For example, the TUG+ test can be seen as a checklist with three questions: Does the patient lie above the TUG and SDa_ap_, or below Da_v_ thresholds? A “diagnostic” of the risk/no risk of fall can be made from these three answers. Both tests could have been designed in a more complex way by resorting to logistic regressions: We present that approach in Appendix A. The performances of both versions of the TUG+ test are equivalent.

### 3.3. AI Classification

**Figure 7:**
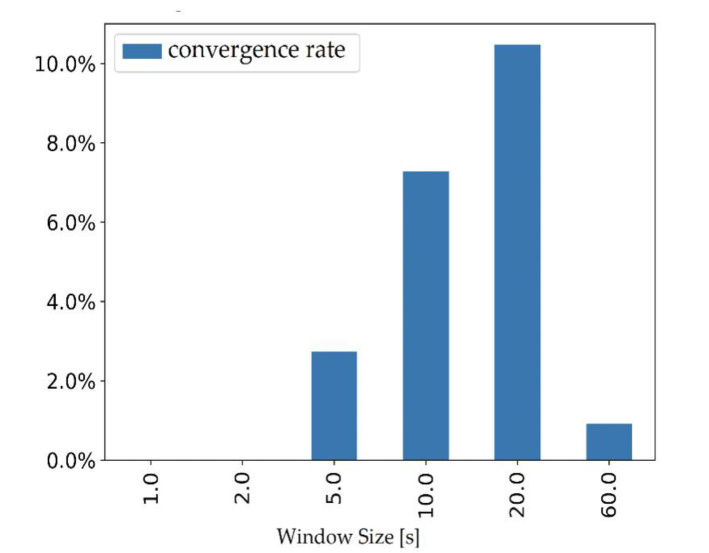
Convergence rate of the AI algorithm versus window size. The convergence rate is the ratio of models that reached a precision of at least 65% on test data and the total amount of models trained for a specific window size.

Models were trained and tested for the following window sizes: 1, 2, 5, 10, 20 and 60 s. Twenty seconds windows showed the best convergence rate as shown in Figure 7. We have then performed a random search hyperparametric study to optimize the accuracy of our AI algorithm [23]. The convergence rate may seem small but it is the consequence of the small (with respect to deep learning) dataset at our disposal and of the wide range of parameters we explored in the parameter space.

**Figure 8:**
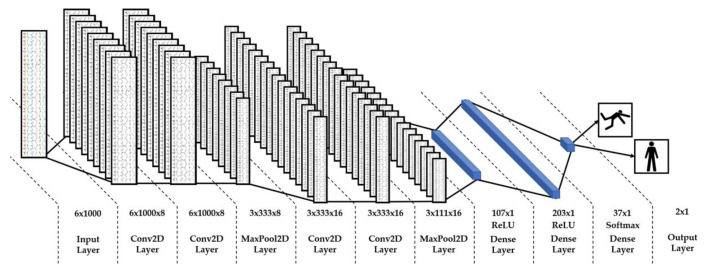
CNN-based AI algorithm used to predict the risk of fall. The input layer receives a 20 s frame from the 6 inputs of the DYSKIMOT sensor. The convolutional layers (Conv2D layer) extract meaningful features from its input and present it to the next stage. Pooling layers (MaxPool2D Layer) reduce the dimension of the feature space by retaining the meaningful inputs of its layer only. After several convolutional stages, the remaining features are injected in a three layers neural network (dense layer) in order to classify the participant’s frames (output layer). The risk of fall is the mean (ranging from 0 to 1) of prediction on all frames of the participant’s sequence. A mean greater than 0.5 denotes a participant with risk of fall.

We have kept the solution presented in Figure 8, leading to the confusion matrix displayed in Figure 6D. It showed maximal accuracy while being equally specific and sensitive (Sp = Se). One solution with higher accuracy was found (Acc = 81%) but at the expense of sensitivity (Se = 62.5%) and was, therefore, not kept.

## 4. Discussion

Our study aimed at designing tests assessing the risk of fall in nursing home patients. The predictions of these tests were compared to the faller/nonfaller status of the participants. The originality of the present work is that two examination times separated by six months were included, and that the fall records of the nursing home during that time was used to classify participants as fallers or nonfallers.

We first confirm that the TUG test alone may predict falls despite its simplicity. We do not observe sensitivity and specificity as high as in [24] (Sp and Se of 87%). This could be explained by the fact that their sample only included 30 participants. Furthermore, a difference of 3.9 s between fallers and nonfallers is observed in our survey, which correlates with that of [25] (difference of 3.59 s). The threshold defining the risk of falls varies between 13 and 32.6 s according to the studies quoted by [26]. Our value of 22.5 s is intermediate. This latter study shows that the TUG test is a test that allows a better division between fallers and nonfallers when they are nursing home residents but not when they are home-based. This conclusion is shared by [26].

Kinematic analysis of the six-minute walking using the DYSKIMOT sensor reveals that this low-cost wearable device is able to measure significant differences between fallers and nonfallers. It is the first time our homemade system is applied to a geriatric population. The homemade sensor we used is not wireless yet. Placing the sensor in the lower limb (e.g., in one shoe) would have been relevant, as it is known from the literature, that instrumented socks are able to identify gait events [27,28], but the wire was uncomfortable for participants. This is coherent with the findings of [29] showing that patients mostly favor devices placed in the upper part of the body. The most comfortable solution we found is the placement in the lumbar region, which leads moreover to a sensor near the participant’s body center of mass, i.e., a crucial point as far as stability and balance are concerned.

The magnitude of anteroposterior acceleration’s fluctuations is significantly larger in fallers. It is coherent with the findings of [30] showing that elderly people who have already fallen have longer deceleration periods during a walking cycle than healthy young people. This behavior aims at reducing the swing phase to shorten unstable periods during the walking cycle. fallers also present smaller fractal dimensions. This observation can be related to the optimal complexity framework of [31]. They argued that physiological signals recorded in a healthy individual have a maximal complexity (e.g., high fractal dimension or entropy). A loss in complexity is associated with aging or disease. For instance, a smaller complexity in the walking pattern of healthy aged participants was observed in [32]. In our population, fallers are less able to perform quick modifications of their walking pattern and show a less complex behavior than nonfallers. Note that the six-minute walking test was interrupted by turnarounds due to the typical 25 m length of the nursing home corridors. Even if in the case of a point-to-point track, a walking course of 30 m is preferred, the minimum recommended length is 15 m [33]. Still, it is worth noticing that turnarounds are complex motor tasks that are representative of the daily activities of nursing home residents. Therefore, the kinematic information contained in the turnarounds could be isolated from the straight gait time series for separate analysis.

According to the review [34], techniques combining sensors and clinical tests are encouraging but the protocols used have yet to be standardized (sensor position, choice of clinical tests, data analysis). It is, therefore, possible to find combinations of clinical tests and kinematic parameters with an accuracy between 47.9% and 100% in a given sample [34]. The choice of the TUG test appears adequate in view of its intensive use in the field of geriatric rehabilitation and simplicity to perform. This test provides important information to predict the risk of fall when combined with kinematic data. The position of the inertial sensor in the lumbar area is relevant as it is close to the body center of mass. Studies such as [35] show that it is at this position that the best information can be gathered in order to differentiate between fallers and nonfallers. Furthermore, our choice of a six-minute walking test with an inertial sensor provides long-term information about an individual’s gait. Such information is not available with the TUG test alone. It appears from our study that the TUG test combined with kinematic parameters such as SCa_ap_ and Da_v_ collected during the six-minute walking test improves the accuracy in predicting falls.

We report here for the first time on the increase of accuracy of TUG+ compared to TUG in predicting falls in elderly nursing home people. The novelty of this TUG+ is to combine TUG stopwatch-based duration results to gait variability indicators measured during a six-minute walking test by the DYSKIMOT sensor. Previous studies followed a similar approach. The study [36] is an attempt to improve the TUG test by increasing the walking distance and by timing each phase of this move (chair lifting, walking time, turnaround time) in order to get more information during the test. Others chose to complement the TUG test with additional sensors in order to improve its effectiveness [37,38]. In [38], the TUG test is specifically coupled with inertial sensors and shows an accuracy of 88%. In another survey, the same authors combined a questionnaire-based clinical evaluation with kinematic data measured by an inertial sensor during the TUG test [37]. They obtained accuracies of 68% for clinical evaluation alone, 73% for inertial sensors alone and 76% for combined evaluation. Those results are similar to ours: We find that the TUG+ test is a better way to predict the risk of falls in the elderly than the TUG test alone: We managed to increase the accuracy of the TUG test by 8.2% (Table 3). Using the TUG+ test, about 74% of the individuals on our sample were correctly categorized. To our knowledge, it is the first time that TUG and six-minute walking test results are merged that way.

We finally have built a complementary approach: AI analysis of six-minute walking kinematical time series in view of predicting the risk of fall. AI techniques are nowadays able to detect falls in real-time [39,40,41]. The particularity of our first attempt is to focus on a six-month prediction rather than on real-time detection. The obvious weakness of our AI classification, based on a convolutional neural network, is the size of our data set. Still, it shows the feasibility of risk of fall prediction from the kinematic data of an elderly walking six minutes, with an accuracy, specificity and sensibility of 75%.

The TUG+ test is an interesting solution in nursing homes because patients generally favor systems that do not replace a therapist [42]. Since our thresholds were fitted on the full population, we can safely conclude that a simple augmented clinical test is able to assess the risk of fall in our sample. The next step in this research is to study a different sample of nursing home patients with the tests built in the present study in order to fully assess our test’s predictive power. We hope to present such results in future work.

AI classification of the risk of fall, combined with a wearable sensor, gives hope that relevant tools monitoring the risk of fall of home-based elderly will become available in the near future. The MARG sensor we used is small (9 cm^2^) and light (10.44 g) enough to imagine several sensors attached on a wearable shirt, as proposed in [16,43] where it is shown that an undershirt equipped with 11 sensors is able to recognize several complex manual material handling tasks and basic postures (sitting, standing and lying down) as well as walking and running. In view of these promising results, it can be hoped that increasing the number of sensors in our system will increase the AI’s accuracy in the assessment of risk of fall. We leave such a program for future works.

## Figures and Tables

**Table 1:** Main characteristics, comorbidities and number of medications increasing the risk of fall of the participants. Fallers were identified according to the fall records between the 6 months interval (t_1_ and t_2_).

	t_1_	t_2_
N	80	73
Age (years)	83.2 ± 8.2	83.0 ± 8.3
Male/Female	28/52	28/45
Walking aid required	49	52
Hypertension (%)	44	42
Number of medications	4 [2–5]	4 [2–5]
Cerebrovascular accident (%)	10	10
Dementia (%)	14	16
Previous heart surgery (%)	21	23
Diabetes (%)	16	15
Hip or knee replacement (%)	16	16
Fallers		23
TUG (s)	20 [17–27]	17 [14–23]

Age is indicated under the form mean ± standard deviation. Medications increasing the risk of fall: psychotrope, antiarrhythmic, diuretics [18]. Timed Up and Go (TUG) and number of medications results are given under the form median [1st quartile–3rd quartile]. Hypertension is defined as a value >140/90 mmHg.

**Table 2:** Comparison between various indices of fallers and nonfallers at t_1_. Data are given under the form median [1st quartile–3rd quartile]. SD = standard deviation, D = fractal dimension. These indices are followed by a subscript labeling the time series from which it was obtained. Results of the TUG test are given in the last line. Significant differences are written in bold font.

	Fallers	Nonfallers	*p*
SDa_v_ (m/s^2^)	0.0949 [0.0810–0.149]	0.101 [0.0868–0.130]	0.245
SDa_ml_ (m/s^2^)	0.0864 [0.0752–0.109]	0.0950 [0.0747–0.109]	0.891
SDa_ap_ (m/s^2^)	0.120 [0.0901–0.173]	0.0900 [0.0753–0.120]	**0.010**
SDω_v_ (°/s)	17.4 [15.5–20.2]	18.4 [15.0–21.9]	0.957
SDω_ml_ (°/s)	15.8 [12.3–20.3]	13.7 [11.1–19.2]	0.480
SDω_ap_ (°/s)	8.29 [6.77–11.6]	8.79 [7.44–12.6]	0.487
Da_v_	1.78 [1.73–1.82]	1.81 [1.77–1.85]	**0.044**
Da_ml_	1.78 [1.66–1.81]	1.81 [1.77–1.83]	0.088
Da_ap_	1.73 [1.68–1.80]	1.79 [1.73–1.83]	0.072
Dω_v_	1.71 [1.67–1.76]	1.74 [1.69–1.76]	0.376
Dω_ml_	1.74 [1.71–1.78]	1.78 [1.72–1.82]	0.098
Dω_ap_	1.81 [1.75–1.83]	1.82 [1.78–1.85]	0.149
TUG (s)	23 [19–31]	19 [16–25]	**0.035**

**Table 3:** Characterization of the TUG and TUG+ test. The following parameters are displayed: sensitivity (Se), specificity (Sp), positive (LR_+_) and negative (LR_−_) likelihood ratios, positive (PPV) and negative (NPV) predictive values and accuracy (Acc). Improvements of TUG+ and AI methods with respect to TUG are emphasized in bold font.

	TUG	TUG+	AI
Se	0.714	**0.857**	**0.750**
Sp	0.541	0.500	**0.750**
LR_+_	1.56	**1.71**	**3.00**
LR_−_	0.529	0.286	0.333
PPV	0.481	**0.778**	**0.750**
NPV	0.761	0.632	0.750
Acc	0.657	**0.739**	**0.750**

**Figure A1:**
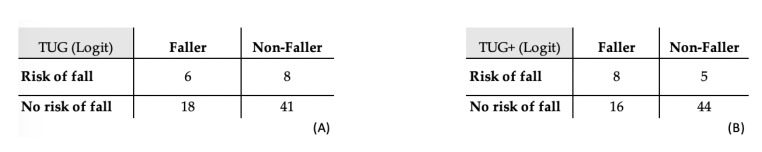
Confusion matrices for the logistic regression-based versions of the TUG and TUG+ tests. (**A**) TUG test. (**B**) TUG+ test.

## Appendix A. TUG and TUG+ Tests with Logistic Regressions

Instead of computing the threshold for TUG time from the ROC curve, a logistic regression of $LOGIT=ln\left(\frac{P}{1-P}\right)$ vs. the TUG time can be performed, P being the probability of being a faller (the R package caret is used). This test, called TUG (Logit), is defined by LOGIT=−1.71+0.0435 TUG(s) and the threshold P* = 0.41; its confusion matrix is presented in Figure A1 and its parameters are given in Table A1. The threshold was fitted to reach optimal accuracy.

**Table A1:** Characterization of the TUG (Logit) and TUG+ (Logit) tests. Values in bold font represent improvements of TUG+ (Logit) test with respect to TUG (Logit) test.

	TUG (Logit)	TUG+ (Logit)
Se	0.837	**0.898**
Sp	0.250	**0.333**
LR_+_	1.12	**1.35**
LR_−_	0.652	0.306
PPV	0.695	**0.733**
NPV	0.439	**0.615**
Acc	0.644	**0.712**

The TUG+ test can also be reformulated by using a logistic regression of LOGIT vs. TUG time, SDa_ap_ and Da_v_. This test, called TUG+ (Logit), is defined by $LOGIT=-3.58+0.0481\;TUG\left(s\right)+7.29\;SDa_{ap}\left(\frac{m}{s^{2}}\right)+0.577\;Da_{v}$. With the same threshold, one is led to the results presented in Figure A1 and Table A1.

The features of TUG (Logit) test are significantly different from TUG test: A Mc Nemar test leads to *p* < 0.01. The TUG test has a higher accuracy than the TUG (Logit) one. However, the performance of TUG+ (Logit) and TUG+ tests are very similar. A Mc Nemar test leads to *p* = 0.149.
